# Commentary: The causal role of gastroesophageal reflux disease in endometriosis: a bidirectional Mendelian randomization study

**DOI:** 10.3389/fmed.2025.1522085

**Published:** 2025-05-16

**Authors:** Bingdi Wei, Mengru Han, Jiali Niu, Shuai Qi, Qing Liu

**Affiliations:** Gansu University of Chinese Medicine, Lanzhou, China

**Keywords:** Mendelian randomization (MR), gastroesophageal reflux disease (GERD), endometriosis, causal inference, genetic variants

## 1 Introduction

Endometriosis is the most common benign gynecological condition, affecting approximately 10% of women and girls of reproductive age worldwide (1). This is a chronic disorder affected by estrogen regulation and presents with symptoms such as dysmenorrhea, pelvic pain, and psychological distress that impose a great social and economic burden on affected individuals (2). This timely diagnosis is further complicated by the overlapping symptoms of intestinal or bladder irritation, thus delaying the identification of the condition (3, 4). At the same time, in connection with gastroesophageal reflux disease (GERD), non-specific symptoms include acid reflux and chronic cough, which create diagnostic confusion with other conditions (5). Clinical observations have pointed out a probable interrelationship between GERD and endometriosis (6), reporting that women with GERD may experience exacerbation of symptoms after endometriosis treatment (7). Although these clinical associations offer exciting prospects, it is very hard to establish a cause-and-effect relationship. Mendelian randomization (MR) will be a new strategy to fill this gap in the literature by using genetic variants as instrumental variables in estimating the question of causality without the confounding inherent in observational studies (8). Discussions by Shi et al. (9) on the cause-and-effect relationship between GERD and endometriosis via MR reveal new insights into the pathogenesis of endometriosis and further improves the early diagnosis and interventional strategy for patients with endometriosis suffering from GERD. This study, however, has certain limitations in some aspects.

## 2 Comments and analysis

### 2.1 Advantages

#### 2.1.1 Innovativeness

This study uses an innovative bidirectional MR analysis to investigate the causal relationship between GERD and endometriosis. MR is a relatively new research approach that uses genetic variants as instrumental variables, helping to mitigate the influence of confounding factors and thereby improving the accuracy of causal inferences. Traditional observational studies often face challenges in establishing causality, whereas MR provides a more rigorous framework for such investigations. This is the first application of MR analysis to the relationship between GERD and endometriosis, paving the way for exploring associations between complex diseases and marking a significant advance in causal inference research.

#### 2.1.2 Methodological rigor

The study employs multiple MR analysis methods—including inverse variance weighting (IVW), MR-Egger regression, and weighted median approaches—to enhance the robustness of causal inferences. By incorporating various methods, the research validates the reliability of its results from different perspectives. It also utilizes multiple sensitivity tests, such as Cochran's Q test, and the MR-PRESSO test, to assess the issues of pleiotropy and heterogeneity. Using these tests strengthens the scientific rigor of the study, helps control potential biases, and ensures the statistical robustness of the findings. This methodological validation provides stronger support for the credibility of the conclusions.

#### 2.1.3 Reliability of data sources

The research data are obtained from large genome-wide association study (GWAS) databases, including the UK Biobank and FinnGen databases in Europe. These databases have undergone strict ethical and data quality reviews, demonstrating high reliability. The large sample size offers a wealth of genetic information, providing statistical support for the analyses and enhancing the efficacy and representativeness of the results. The credibility of the data sources not only boosts the rigor of the study but also increases the applicability of its conclusions in European populations.

### 2.2 Limitations

Although bidirectional MR analysis was used, the lack of additional methods for result verification limits the robustness of causal inferences. In the reverse causal analysis, the small sample size and insufficient number of effective instrumental variables single nucleotide polymorphisms (SNPs), especially in the analysis of localized endometriosis, which only included five SNPs, diminish the statistical power of the reverse analysis results. Furthermore, the study did not investigate the dose–response relationship between the severity or duration of GERD and endometriosis, leading to a somewhat one-sided understanding of the causal relationship.

#### 2.2.1 Inadequacies in data analysis

The study relies on *p*-values to determine statistical significance without a detailed interpretation of effect sizes and confidence intervals. Although the association between GERD and endometriosis is statistically significant (OR = 1.47, *P* = 0.05), the effect size is small and the confidence interval approaches 1, indicating a weak actual impact (10). Additionally, the insufficient effectiveness of the instrumental variables in the reverse MR analysis may lead to weak instrument bias, affecting the accuracy of causal inferences. The study also inadequately controls for pleiotropy and heterogeneity, increasing the risk of bias in data analysis.

#### 2.2.2 Limitations in sample sources

While the study utilizes high-quality European datasets (UK Biobank and FinnGen), the limited ethnic diversity of these samples raises important concerns about the generalizability of findings. Significant interethnic variations exist in both disease prevalence (GERD: 18.1%−27.8% in European populations vs. 2.5%−7.8% in East Asian cohorts) and genetic architecture (e.g., differential effect sizes for risk loci such as rs1799964) (11). To address this limitation in future research, we recommend (1) incorporating trans-ethnic GWAS consortia (e.g., Biobank Japan, China Kadoorie Biobank) with standardized phenotyping protocols, (2) implementing genetic ancestry principal components as covariates to account for population stratification, and (3) conducting stratified analyses by key demographic variables (age tertiles, body mass index categories, and menopausal status) to evaluate effect heterogeneity (12, 13). Such approaches would enable differentiation between genetically driven and environmentally mediated mechanisms while improving the clinical applicability of findings across diverse populations.

#### 2.2.3 Logical contradictions in causal inference

The results of the forward and reverse MR analyses are inconsistent. The forward MR analysis indicates that GERD may increase the risk of endometriosis, while the reverse analysis shows no significant impact of endometriosis on GERD. This unidirectional causal relationship lacks biological support and does not adequately explain why GERD affects endometriosis without reciprocal effects. This logical contradiction remains unresolved, undermining the scientific rigor of the study.

#### 2.2.4 Insufficient biological plausibility

While this study establishes a statistically significant association between GERD and endometriosis (OR = 1.47, *P* = 0.05), the biological mechanisms underlying this relationship remain insufficiently explored. Current evidence suggests multiple potential pathways: (1) GERD-induced gastric acid reflux may promote chronic systemic inflammation, with elevated cytokines (IL-6 and TNF-α) activating pelvic macrophages and facilitating ectopic endometrial growth (3, 6); (2) acid reflux-associated gut microbiota dysbiosis (14) and subsequent immune dysregulation may disrupt the gut–endometrial axis, creating a bidirectional pathological loop (15); and (3) vagus nerve activation by chronic reflux could alter the uterine microenvironment through neurogenic inflammation (16). However, the modest effect size and the lack of mechanistic validation limit clinical interpretation. Future research should employ liquid biopsies to track inflammatory markers and animal models to experimentally verify these pathways, particularly focusing on the microbiome–immune interface and neuroendocrine crosstalk.

### 2.3 Impact on the field

This study pioneers the use of bidirectional MR to investigate the causal relationship between GERD and endometriosis, utilizing large-scale GWAS data from European populations. The methodological rigor, including IVW, MR-Egger, and sensitivity analyses, enhances the reliability of causal inferences.

Limitations include the lack of ethnic diversity in samples, potential weak instrument bias in reverse MR analysis, and insufficient exploration of biological mechanisms. While the study provides novel insights, its clinical applicability is constrained by small effect sizes (OR = 1.47) and unresolved bidirectional inconsistency. Future research should integrate multi-ethnic cohorts (e.g., Asian or African populations) and experimental models to validate these findings, as suggested by recent microbiome studies (14, 15).

### 2.4 Improvement suggestions

#### 2.4.1 Validation with additional methods

To strengthen causal inference, future studies should implement a multi-method validation framework that combines MR with complementary approaches. First, Bayesian MR methods should be used to quantify posterior probabilities of causal effects while incorporating prior biological knowledge about GERD-endometriosis pathways. Second, sensitivity analyses using different pleiotropy-robust methods (e.g., weighted median and MR-PRESSO) should be systematically compared through heterogeneity metrics (*I*^2^ < 25% indicating consistency). For dose–response evaluation, researchers should (1) stratify GERD exposure by clinically validated severity indices (e.g., Los Angeles classification grades) and treatment duration and (2) apply non-linear MR techniques to detect potential threshold effects. This integrated approach would address method-specific assumptions while providing a more nuanced understanding of the exposure–outcome relationship.

#### 2.4.2 Emphasizing interpretation of effect sizes and confidence intervals

Future data analyses should place greater emphasis on the size of effects and confidence intervals to avoid overinterpreting small but statistically significant effects, while ensuring the rigor of causal inferences.

#### 2.4.3 Expanding ethnic diversity in samples

Incorporating diverse ethnic samples will help improve the generalizability of the conclusions and uncover potential differences in causal relationships across different races, providing further support for personalized medicine.

#### 2.4.4 In-depth exploration of biological mechanisms

Future research should investigate the biological mechanisms linking GERD and endometriosis through three key pathways: (1) microbiome–immune interactions, where GERD-induced dysbiosis may promote endometrial inflammation (14); (2) neuroendocrine pathways mediated by vagus nerve signaling (16); and (3) systemic inflammation involving elevated cytokines (IL-1β, IL-6, and TNF-α) that enhance endometrial adhesion and angiogenesis. These mechanisms should be explored using multi-omics approaches, animal models, and liquid biopsy techniques (16).

#### 2.4.5 Incorporating mediating variable analysis

Future mediation analyses should employ a rigorous two-step MR approach to investigate psychological and biological mediators. Key steps include (1) identifying candidate mediators through genetic correlation analyses between psychiatric traits (e.g., depression GWAS) and disease endpoints and (2) quantifying mediation effects using instrumental variables for both exposure-mediator and mediator-outcome pathways. Particular focus should be given to Hypothalamic-Pituitary-Adrenal axis-related mediators (e.g., NR3C1 polymorphisms), with mediation effects considered significant only when demonstrating ≥20% attenuation of the primary association after adjustment. This approach maintains biological plausibility while providing clinically interpretable effect estimates.

### 2.5 A unified roadmap for causal translation

To operationalize these improvements, we propose a translational pipeline: (1) Discovery Phase: trans-ethnic MR with Bayesian False Discovery Rate (FDR) control; (2) Mechanistic Phase: multi-omics mediation (MENA + organoids); (3) Clinical Phase: target prioritization via Population Attributable Fraction (PAF) and Number Needed to Treat (NNT)-based cost-effectiveness analysis. This framework explicitly links genetic findings to clinical actionability while addressing all reviewer concerns through measurable benchmarks (e.g., FDR < 0.05, PAF > 10%, and NNT < 20).

## 3 Conclusion

This study, through MR methods, offers a new exploratory pathway for the association between GERD and endometriosis. However, limitations in methodology, sample diversity, and biological explanation hinder a clear distinction between correlation and causation. If future research can optimize the aspects of methods, samples, and mechanisms, it will provide more persuasive evidence for the study of associations among complex diseases and promote the development of this field.
